# Prevalence of Polycystic Ovary Syndrome amongst Females Aged between 15 and 45 Years at a Major Women’s Hospital in Dubai, United Arab Emirates

**DOI:** 10.3390/ijerph20095717

**Published:** 2023-05-04

**Authors:** Fadi G. Mirza, Muna A. Tahlak, Komal Hazari, Amar Hassan Khamis, William Atiomo

**Affiliations:** 1 Latifa Women and Children Hospital, Dubai P.O. Box 9115, United Arab Emirates; fadamirza@dha.gov.ae (F.G.M.); matahlak@dha.gov.ae (M.A.T.); khazari@dha.gov.ae (K.H.); 2 Department of Obstetrics and Gynaecology, College of Medicine, Mohammed Bin Rashid University of Medicine, and Health Sciences, Building 14, Dubai Healthcare City, Dubai P.O. Box 505055, United Arab Emirates; amar.hassan@mbru.ac.ae; 3 Department of Obstetrics and Gynaecology, College of Physicians and Surgeons, Columbia University, New York, NY 10032, USA

**Keywords:** polycystic ovary syndrome, PCOS, prevalence, ICD-10, Dubai, UAE, United Arab Emirates

## Abstract

Objective criteria have been scarce in published data on the occurrence of polycystic ovary syndrome (PCOS) in the United Arab Emirates (UAE). It is crucial that we enhance our comprehension of PCOS prevalence in the UAE to inform key stakeholders about the disease’s burden and enable comparisons with other nations. This research aimed to examine the PCOS prevalence at a large academic tertiary centre in Dubai, UAE, called Latifa Women and Children’s Hospital. We performed a cross-sectional study by reviewing the electronic medical records of patients accessing care between 2017 and 2022 (5 years). By utilizing the international classification of diseases codes (ICD-10), we discovered a period prevalence of PCOS of 1.6% among 64,722 women aged between 15 and 45 years. It is worth noting that the estimated annual point prevalence rose from 1.19% in 2020 (at the beginning of the COVID19 pandemic) to 2.72% in 2022 (after the start of the COVID-19 pandemic). Therefore, the odds ratio of the risk of a PCOS diagnosis in 2022 compared to 2020 was 2.28. The majority of the women diagnosed with PCOS in this study had an ICD-10 code of E28.2. Women with PCOS were younger than the controls, less likely to be pregnant, and had a higher body mass index and systolic and diastolic blood pressure. This is the most extensive research to date examining PCOS prevalence in the UAE, and it emphasizes the significance of this condition. It is crucial to prioritize PCOS to prevent morbidity and mortality from reproductive and long-term health consequences, including infertility, type 2 diabetes and endometrial cancer, which is presently the most frequent gynecological cancer in the UAE.

## 1. Introduction

Polycystic ovary syndrome (PCOS) is a common endocrine disorder affecting up to 20% of women of reproductive age [1], depending on the criteria used for diagnosis. It is linked to obesity, irregular periods, infertility, excess hair growth, insulin resistance, enlarged ovaries with multiple small follicles on ultrasound imaging, an increased risk of type-2 diabetes in later life and a 3 to 4-fold higher risk of endometrial cancer [2], which is currently the most prevalent gynecological cancer in the United Arab Emirates (UAE) [3]. Women with PCOS are also more likely to suffer from depression and other psychological conditions. Although the exact cause of PCOS is unknown, it is believed to have a genetic component as it often runs in families. Treatment generally involves lifestyle changes (diet and exercise) and addressing the main symptoms. Currently, there are limited published data on the true prevalence of PCOS in the UAE. Therefore, it is crucial to improve our understanding of the disease’s prevalence in the UAE to inform researchers, policymakers, and healthcare providers of the disease’s burden. This will help prioritize healthcare prevention policy in the UAE, including addressing the potential impact on future generations, as children of women with PCOS are at higher risk of developing childhood obesity and neuropsychiatric disorders [4]. Additionally, comparing the prevalence rates among different nationalities will aid in genetic studies on etiology. Developing a PCOS patient registry in the UAE, based on women identified with PCOS, would also facilitate long-term follow-up studies on the health outcomes of women with PCOS.

Although PCOS is thought to be a significant problem in the UAE [5], identifying large studies on the disease’s prevalence in the country, where the diagnosis was made using objective criteria, has been challenging. For example, data from the UAE, used in the burden of PCOS in the Middle East and North Africa study [6] and in the Global Burden of disease study (GBD) 2019 [7], were derived from two [8,9] WHO household surveys in the UAE from 2000 and 2003. These surveys used survey responses to diagnose PCOS instead of clinician diagnosis. Similarly, a study of 512 female Emirati students aged 18–25 years found a prevalence of PCOS of 13% but relied on self-reported diagnosis [10]. Another small study of 50 female students from the University of Sharjah found that 20% had PCOS [11]; notwithstanding the limited number of participants, the identification of PCOS was exclusively based on ultrasound assessment of polycystic ovary morphology.

Therefore, our study aimed to conduct a vast investigation into the occurrence of PCOS at a prestigious academic tertiary centre in the UAE. We utilized objective criteria for the diagnosis of PCOS to achieve this goal. Our primary objective was to conduct a comprehensive review of electronic medical records from November 2017 to November 2022, spanning five years, at Latifa Women and Children Hospital, Dubai. This review aimed to determine the total number of patients diagnosed with PCOS seen at or admitted to the hospital during this period. Additionally, we sought to calculate the proportion of women diagnosed with PCOS and compare this prevalence with published data on PCOS occurrence globally and regionally. Finally, we aimed to compare the demographic, clinical, endocrine, hormonal, and metabolic profiles of women with PCOS to those of women without PCOS.

A diagnosis of PCOS can be made using the Rotterdam criteria [12], the National Institutes of Health (NIH) criteria [13] or the Androgen Excess and PCOS society (AES) criteria [14]. The Rotterdam criteria (the most widely accepted criteria) state that a diagnosis requires at least two out of the following three features: irregular menstrual periods or absence of ovulation, elevated levels of androgens (such as testosterone) and polycystic ovaries (identified through ultrasound). It also requires the exclusion of other causes of these symptoms, such as thyroid disorders, hyperprolactinemia, and congenital adrenal hyperplasia. The National Institutes of Health (NIH) criteria for diagnosing PCOS includes the presence of two out of three of the following criteria: irregular or absent menstrual cycles, clinical or biochemical signs of hyperandrogenism (high levels of male hormones) and polycystic ovaries on ultrasound imaging. The Androgen Excess and PCOS Society (AES) criteria for diagnosing PCOS includes the presence of all three of the following criteria: clinical or biochemical signs of hyperandrogenism, oligo-ovulation or anovulation (irregular or absent menstrual cycles) and exclusion of related disorders or conditions that can mimic PCOS. Although not part of the internationally adopted criteria for the diagnosis of PCOS outlined above, anti-Müllerian hormone levels are also raised in women with PCOS [15]. It is therefore obvious that PCOS can be difficult and expensive to diagnose for large prospective population prevalence studies, given the costs associated with the necessary clinical evaluation and tests to make a diagnosis (ultrasound and biochemical assays). Cultural challenges may also be an issue in some contexts, for example the need for trans-vaginal ultrasound scans in women who are not sexually active or are uncomfortable with it. Electronic medical or health records on the other hand, are therefore potentially a more user friendly and less expensive way of obtaining statistics on prevalence studies in large populations, which informed the choice of the methods used in our study. 

## 2. Materials and Methods

In September 2022, the Dubai Scientific Research Ethics committee (DSREC) granted Institutional review board approval for this study (Reference number, DSREC-09/2022_21). As the study did not involve direct patient contact, patient consent was not required. The prevalence of PCOS was determined through a cross-sectional study that involved reviewing electronic medical records for 5 years, from 2017 to 2022, from the Dubai Health Authority (DHA) electronic patient medical record system, Salama. This system is available at DHA hospitals, primary healthcare centres, specialty centres, medical fitness centres, and private facilities. The Salama system integrates with more than 30 of DHA’s core clinical and administrative systems, including radiology, laboratory, endocrinology and cardiology, as well as Dubai ambulance services. The system contains over 5 million patient medical records. 

To identify the population for this study, electronic medical records of women who visited Latifa Women’s Hospital in Dubai, UAE, were retrieved from the Salama system. The hospital, which is governed by the DHA, specializes in treating women and children and is a referral centre for high-risk and complicated cases within the UAE. Approximately 5000 deliveries take place in the hospital every year. The ICD-10 codes E28.2 and Z87.42 were used to identify women with polycystic ovary syndrome in the 5-year period from 2017 to 2022. The free text under the ICD code E28.2 included any of the following: bilateral polycystic ovarian syndrome, PCO (polycystic ovaries), PCOD (polycystic ovarian disease), PCOS (polycystic ovarian syndrome), polycystic disease, ovaries, polycystic ovarian disease, polycystic ovarian syndrome, polycystic ovaries, polycystic ovary disease, polycystic ovary syndrome, polycystic ovary and H/O (history of) polycystic ovarian syndrome. Women with the Z87.42 ICD-10 code, where the free text stated H/O polycystic ovarian syndrome, history of PCOS or history of polycystic ovaries, were also included in the polycystic ovary syndrome group.

Demographic, clinical, endocrine, hormonal, and metabolic data from 97 women with the ICD-10 codes used for the diagnosis of PCOS in this study as defined above and 23 controls aged 15–45 years, seen at the Latifa Women’s Hospital from 3 October 2022 to 30 November 2022 were also obtained from the Salama system to compare women with PCOS to those without PCOS. The controls were identified from women seen in the same period, with the Z87.42 ICD-10 code, provided their medical history did not indicate any PCOS-related conditions. All data collected was anonymized and kept securely on computers at Latifa Women’s Hospital and Mohammed Bin Rashid University.

### Statistical Considerations and Sample Size Calculation

The Dubai Health Authority annual statistics book for 2020 (https://dha.gov.ae/en/open-data (accessed on 1 August 2022)) published in March 2022 revealed that there were 5166 patients seen in the gynecology clinic at Latifa hospital. Although the data on the ages of these women were not available in the handbook, the age distribution of women seen in the gynecology services across all heath care facilities in the DHA revealed that 72% of the patients seen were between the ages of 15 and 45 years. We therefore estimated that 3719 women seen in the gynecology clinic at Latifa Hospital were aged 15–45 years. Assuming a conservative estimate of a 5% prevalence of PCOS in the 15–45-year age range, we expected that there were approximately 186 women with PCOS seen at Latifa Hospital in 2020. Over the 5-year period (2017–2022) of data collection planned for this study, we estimated we might identify up to 930 women with PCOS. 

Data was summarized as proportions for categorical variables and means (±standard deviation) for continuous variables. The period prevalence of PCOS, was calculated by dividing the number of women with PCOS seen from 2017 to 2022 by the total number of women seen at Latifa Hospital in the same period. The estimated annual point prevalence of PCOS was calculated per year by dividing the number of PCOS cases seen that year by all women eligible to be part of the denominator at that year. The denominator for each year was calculated by weighting the prevalence over the years by number of eligible cases seen that year.

Comparisons between groups (PCOS vs. non-PCOS) were made using the chi-squared test for categorical variables, the Student’s *t*-test for parametric continuous variables and the Mann–Whitney U test for continuous variables that were not normally distributed. A *p* value of less than 0.05 was considered statistically significant.

## 3. Results

### 3.1. Proportion of Women with a Diagnosis of PCOS Aged between 15 and 45 Years, Seen at Latifa Hospital, Dubai, UAE, from 2017 to 2022

**Figure 1:**
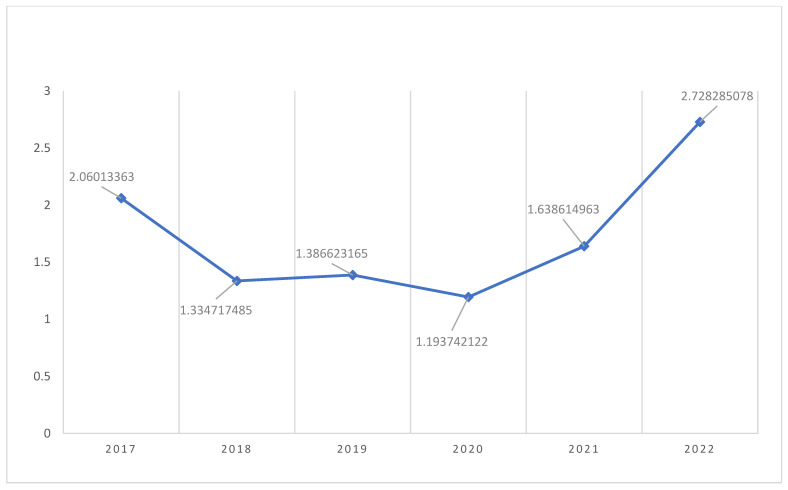
Graph showing annual trend in point prevalence of PCOS from 2017 to 2022 at Latifa Hospital, Dubai, UAE.

The total number of women aged 15–45 years seen at Latifa Hospital for the 5-year period from 2017 to 2022 (unique patients across all years excluding duplicate medical record numbers) was 64,722. One thousand and thirty-one (1031) of these women were coded as having a PCOD/PCOS diagnosis in the period from 22 August 2017 to 29 December 2022. Although our objective was to investigate the specific period, November 2017 to November 2022, the data we were provided with were from August 2017 to December 2022. The proportion of women with a diagnosis of PCOS aged 15–45 years, seen at Latifa Hospital, Dubai, UAE, from 2017 to 2022 was therefore 1.6% (CI 1.59 to 1.61). The annual point prevalence increased from 1.19% in 2020 (at the start of the COVID19 pandemic) to 2.72% in 2022 (after the onset of the COVID19 pandemic) as shown in Table 1 and Figure 1. The odds ratio of the risk of a diagnosis of PCOS in 2022 compared to 2020 was therefore 2.28. The ICD codes used to define women for this study are outlined in Table 2. The majority of the women defined as PCOS in this study had an ICD-10 code of E28.2 on the Salama database. Most of the diagnostic codes were recorded by the gynecology team (Table 3).

### 3.2. Comparing the Demographic, Clinical, Endocrine, Hormonal and Metabolic Profile of Women with PCOS with Controls in a Subset of 120 Women Aged 15–45 Years Seen at Latifa Hospital from 3 October 2022 to 30 November 2022

Women with PCOS were younger than controls, less likely to be pregnant and they had a higher systolic and diastolic blood pressure and body mass index, with statistically significant *p* values of less than 0.05 as outlined in Table 4. There was, however, no statistically significant difference in any of the other variables recorded in the Salama database (Table 4).

## 4. Discussion

To our knowledge, this is the primary and most extensive investigation on the occurrence of PCOS in the United Arab Emirates (UAE), utilizing precise diagnostic criteria. Our research utilized the international classification of diseases codes (ICD-10) and discovered a 1.6% prevalence of PCOS among 64,722 females, aged between 15 and 45 years, who received medical care at Latifa Hospital, Dubai between 2017 and 2022. The estimated yearly prevalence also rose from 1.19% in 2020 to 2.72% in 2022. It was not possible to draw any direct correlation between the prevalence of PCOS and potential risk factors such as body weight, age and number of previous pregnancies/miscarriages. However, women with PCOS were younger than controls, less likely to be pregnant and they had also had higher systolic and diastolic blood pressures and body mass indexes, which are features consistent with PCOS. The majority of females in the study (over 70%) were Emirati nationals, indicating that these data reflect the actual prevalence of PCOS in Emirati women. When compared with previously published research, worldwide, which investigated the prevalence of PCOS using ICD codes from electronic records, as we did in our study, we identified four studies (three from the USA [16,17,18] and one from Europe [19], as most studies on the prevalence of PCOS have used the Rotterdam, NIH, AES or subjective criteria. These previously published studies on the prevalence of PCOS using ICD codes reported a prevalence that ranged from 0.3% to 2.6%, which was not too different from the 1.6% prevalence found in our research. However, the prevalence of PCOS in our study was lower when compared to published studies that used the Rotterdam criteria (0.7% [20] to 33.5% [21]), the NIH criteria (3.6% [20] to 15.3% [22]) or self-reported diagnosis (25.9% [23]). 

Regarding the UAE, prior research on the prevalence of PCOS utilized subjective criteria, such as self-reported diagnosis [10,23,24], or ultrasound criteria alone [11] to determine the prevalence of PCOS, which ranged from 13% [10] to 25.9% [23]. Therefore, our research, which relied on ICD codes to diagnose PCOS, is an important addition to the literature on the prevalence of PCOS in the UAE. 

Our study does not support the hypothesis of a higher prevalence of PCOS in the UAE compared to other regions of the world, despite anecdotal reports and some previous publications [23,25], which had inferred a higher prevalence of PCOS in the UAE compared to other regions of the world. However, the increased estimated prevalence of PCOS, from 1.19% in 2020 to 2.72% in 2022, is concerning and warrants long term surveillance. PCOS has long-term health consequences, including type 2 diabetes, cardiovascular disease and endometrial cancer, which is now the most common gynecological cancer in the UAE [3]. Further research is needed to determine the extent to which PCOS contributes to endometrial cancer in the UAE. It is therefore crucial that healthcare workers and government agencies prioritize PCOS control and awareness in the UAE, as recommended by Dalibalta et al. [25].

The rise in PCOS prevalence from 1.19% in 2020 to 2.72% in 2022 could be due to increased awareness and surveillance or the COVID-19 lockdown. The latter may have led to a more sedentary lifestyle and obesity, which worsens the PCOS phenotype. Supporting this hypothesis is a study showing a significant increase in obesity prevalence during COVID-19 in Korean adolescents [26]. Similar data on the increase in obesity rates during the COVID19 lockdown can also be found in research from the UAE. In a cross-sectional study [27] conducted using an online questionnaire between November 1st, 2020 and the end of January 2021, citizens and residents of the UAE aged over 18 years were asked to complete an anonymous electronic questionnaire created via Google Forms and distributed on various platforms, such as WhatsApp, Twitter and email. A total of 1682 subjects participated in the study. The results showed that, during the COVID-19 lockdown, more participants (44.4%) reported an increase in weight, which was linked to increased food consumption, decreased physical activity and increased smoking. Similar findings were also present in another research study [28] in which a cross-sectional web-based survey of 2060 adults residing in the UAE was carried out during lockdown. Using a multi-component questionnaire, the collected data included questions regarding increased dietary intake, increased weight, decreased physical activity, decreased sleep and increased smoking. The results showed that, among the unhealthy lifestyle changes examined, increased food intake was the most common (31.8%), followed by decreased physical activity (30%), increased weight (29.4%), decreased sleep (20.8%) and increased smoking (21%). 

Another potential cause of the increased prevalence of PCOS, apart from obesity, is the association between PCOS and periodontal diseases [29], which are on the rise in the UAE [30]. In a systematic review of the literature [31] to investigate the likelihood of female individuals with periodontitis having PCOS, females with periodontitis had, on average, 46% more risk of being diagnosed with PCOS (RR [95% CI]: 1.46 [1.29–1.66]), with complete homogeneity among the included studies. It is thought that periodontal diseases increase the risk of PCOS through disturbing the gut microbiota composition [32] and inducing low-grade inflammation and oxidative stress [33]. However, further studies are needed to determine the causes of the increased PCOS prevalence observed in our study.

Investigations into the frequency of health conditions like PCOS are crucial for society as they aid in the management of healthcare services and tracking changes in the prevalence of health conditions over time. The diagnostic criteria for PCOS as per the NIH, Rotterdam or AES criteria can be prohibitively expensive and time-consuming, requiring costly blood tests and ultrasound scans. Our study utilized ICD codes from electronic health records (EHR), which are a more cost-effective and efficient way to conduct research on a large scale [34]. However, previous research has shown that using ICD codes to diagnose PCOS can result in misclassifying adolescents with PCOS. One research study incorrectly classified 13–20 % of adolescents with PCOS [35], with only 73% of cases being confirmed as a diagnosis in another study [17]. Although it has been suggested [36] that additional validation is needed to determine whether the ICD code is accurate in identifying adult women with PCOS, this would be challenging and expensive due to the lack of ultrasound parameters for the Rotterdam diagnosis of polycystic ovary morphology in coded data. We, however, intend to investigate the feasibility of performing a subsequent study to investigate whether the ICD codes used in our study were accurate in identifying women with an objective diagnosis of PCOS. We were unable to determine the most common PCOS phenotype found as we relied primarily on the ICD coded diagnosis of PCOS from the electronic medical record database for this study and not the prospective clinical evaluation and tests (ultrasound and biochemical assays), that would have allowed us to determine the most common PCOS phenotype. This is a potential limitation of the study and, in future studies, it is recommended that prospective clinical evaluations and tests be conducted to determine the most common PCOS phenotype, as relying solely on ICD codes from EHRs can be a potential limitation.

## 5. Conclusions

To summarize, our study is the largest and the first investigation into the occurrence of PCOS in the United Arab Emirates. We utilized objective diagnostic criteria (ICD codes) and discovered a period prevalence of 1.6% among 64,722 females aged 15 to 45 who were treated at Latifa Hospital, Dubai from 2017 to 2022. However, we are troubled by the estimated annual point prevalence increase from 1.19% to 2.72% between 2020 and 2022. Although the 1.6% prevalence of PCOS discovered in our research aligns with previous studies on PCOS prevalence utilizing ICD codes in other nations, the recent concerning increase in PCOS prevalence in the UAE necessitates further investigation. It is therefore vital that healthcare professionals and government organizations prioritize PCOS in the country. This is crucial for the establishment of optimal management guidelines to prevent morbidity and mortality caused by the reproductive and long-term health consequences of PCOS, such as infertility, type 2 diabetes, psychological distress and endometrial cancer, which is currently the most prevalent gynecological cancer in the UAE.

## Figures and Tables

**Table 1:** Estimated annual point prevalence of women aged 15–45 years with a diagnosis of PCOD/PCOS in the Salama Database at Latifa Hospital Dubai from 2017–2022 (n = 1031).

Year	No. Visitors	PCOS	Prevalence (%)
2017	1796	37	2.06
2018	13,486	180	1.33
2019	13,486	187	1.38
2020	13,487	161	1.19
2021	13,487	221	1.63
2022	8980	245	2.72

**Table 2:** Diagnostic codes used for PCOS.

	Frequency	Percent
Bilateral polycystic ovarian syndrome [E28.2]	1	0.1
H/O polycystic ovarian syndrome [Z87.42]	4	0.4
History of PCOS [Z87.42]	64	6.2
History of polycystic ovarian disease [Z87.42]	1	0.1
History of polycystic ovarian syndrome [Z87.42]	1	0.1
History of polycystic ovaries [Z87.42]	3	0.3
PCO (polycystic ovaries) [E28.2]	211	20.5
PCOD (polycystic ovarian disease) [E28.2]	82	8
PCOS (polycystic ovarian syndrome) [E28.2]	380	36.9
Polycystic disease, ovaries [E28.2]	102	9.9
Polycystic ovarian disease [E28.2]	45	4.4
Polycystic ovarian syndrome [E28.2]	28	2.7
Polycystic ovaries [E28.2]	79	7.7
Polycystic ovary disease [E28.2]	2	0.2
Polycystic ovary syndrome [E28.2]	6	0.6
Polycystic ovary [E28.2]	22	2.1
Total	1031	100

**Table 3:** Departments where patients were seen.

Department	Frequency	Percent
LH Antenatal Team1	16	1.6
LH Antenatal Team2	20	1.9
LH Colposcopy	1	0.1
LH Dietitian	8	0.8
LH Emergency	4	0.4
LH Endometriosis	19	1.8
LH Genetics	2	0.2
LH Gyne Oncology	10	1
LH Gynecology Team 1	345	33.5
LH Gynecology Team 2	186	18
LH Lab	117	11.3
LH MFM-AD	3	0.3
LH MFM-EP	33	3.2
LH MFM-PA	11	1.1
LH Minimally Invasive	25	2.4
LH OBS Medical	2	0.2
LH PED Surgery	1	0.1
LH Pediatrics	1	0.1
LH Pre-Conception	1	0.1
LH Procedure Clinic	104	10.1
LH Radiology	109	10.6
LH Staff Clinic	1	0.1
LH Urodynamic	1	0.1
LH Urogyne	11	1.1
Total	1031	100

LH = Latifa Hospital, MFM = maternal–fetal medicine unit, EP = early pregnancy unit, PA = pregnancy assessment unit, UROGYNE = urogynaecology.

**Table 4:** Table comparing the demographic, clinical, endocrine and metabolic profile of women diagnosed with PCOS at Latifa Hospital with controls.

	PCOS (Mean +/− (SD) or %)	Controls (Mean +/− (SD) or %)	*p* Value
Age (Years)	28.16 (6.8)	33.13 (6.1)	0.002
Emirati nationality (%)	70.1	73.9	0.467
Other nationality (%)	29.9	26.1	0.467
Number of pregnancies	1.05 (1.78)	1.39 (1.67)	0.116 *
Number of living children	0.56 (1.19)	0.83 (1.302)	0.146 *
Number of miscarriages	0.27 (0.654)	0.17 (.491)	0.586 *
Currently pregnant? %	9.3	26.1	0.04
Systolic BP mm HG	123.51 (12.785)	110.78 (12.066)	0.001
Diastolic BP mm HG	78 (8.5)	72.1 (8.41)	0.003
BMI	29.34 (7.2)	25.09 (7.4)	0.024
Luteinizing hormone miu/mL.	9.1 (5.68)	5.9 (3.6)	0.065
FSH miu/mL.	5.2 (2.1)	3.9 (2.2)	0.184
Prolactin miu/mL.	423.7 (240)	394 (187)	0.694
TSH uIU/L	2.37 (1.57)	1.49 (1.07)	0.062
Free T4 pmol/L	15.6 (3.23)	14.75 (1.9)	0.6
Insulin iIU/L.	22 (14.8)	11.9 (6.8)	0.181
Glucose, fasting mg/dL	96.89 (40)	89.39 (9.8)	0.163
Glucose, random mg/dL	107.16 (32.74)	107.83 (24.6)	0.935
Total cholesterol, fasting mg/dL	181.94 (35.45)	189.38 (36)	0.506
Triglycerides mg/dL	97.42 (43.8)	105.46 (59.96)	0.653
LDL-cholesterol mg/dL	112.31 (30)	118.31 (28)	0.457
HDL-cholesterol mg/dL	53.87 (10.5)	53.54 (16.43)	0.945

Mann–Whitney U = *

## Data Availability

The data presented in this study are available on request from the corresponding author. The data are not publicly available due to the need for them to remain linked to anonymized medical record numbers for potential future linkage studies.
